# Diverting the Flux of the JA Pathway in *Nicotiana attenuata* Compromises the Plant's Defense Metabolism and Fitness in Nature and Glasshouse

**DOI:** 10.1371/journal.pone.0025925

**Published:** 2011-10-10

**Authors:** Michael Stitz, Ian T. Baldwin, Emmanuel Gaquerel

**Affiliations:** Max Planck Institute for Chemical Ecology, Department of Molecular Ecology, Jena, Germany; USDA-ARS, United States of America

## Abstract

A plant's inducible defenses against herbivores as well as certain developmental processes are known to be controlled by the jasmonic acid (JA) pathway. We have previously shown that ectopically expressing *Arabidopsis thaliana JA O-methyltransferase* in *Nicotiana attenuata* (35S-*jmt*) strongly reduces the herbivory-elicited jasmonate bursts by acting as metabolic sink that redirects free JA towards methylation; here we examine the consequences of this metabolic sink on *N. attenuata's* secondary metabolism and performance in nature. In the glasshouse, 35S-*jmt* plants produced fewer seed capsules due to shorter floral styles, which could be restored to wild type (WT) levels after hand-pollination, and were more susceptible to *Manduca sexta* larvae attack. When transplanted into the Great Basin Desert in Utah, 35S-*jmt* plants grew as well as WT empty vector, but were highly attacked by native herbivores of different feeding guilds: leaf chewers, miners, and single cell feeders. This greater susceptibility was strongly associated with reduced emissions of volatile organic compounds (hexenylesters, monoterpenes and sesquiterpenes) and profound alterations in the production of direct defenses (trypsin proteinase inhibitors [TPI], nicotine, diterpene glycosides [DTGs] and phenylpropanoid-polyamine conjugates) as revealed by a combination of targeted and metabolomics analyses of field collected samples. Complementation experiments with JA-Ile, whose formation is outcompeted in 35S-*jmt* plants by the methylation reaction, restored the local TPI activation to WT levels and partially complemented nicotine and DTG levels in elicited but not systemic leaves. These findings demonstrate that MeJA, the major JA metabolite in 35S-*jmt* plants, is not an active signal in defense activation and highlights the value of creating JA sinks to disrupt JA signaling, without interrupting the complete octadecanoid pathway, in order to investigate the regulation of plants' defense metabolism in nature.

## Introduction

In response to the attack of various arthropod herbivores, plants reconfigure their metabolism and synthesize a broad set of phytochemicals that can either act as toxins or as digestibility reducers [1]. The wild tobacco, *Nicotiana attenuata*, is equipped with a suite of inducible metabolites whose regulation and impact on the plant's resistance to different herbivores have been extensively studied in laboratory and field conditions. The best characterized and most active of these direct defense compounds are the neurotoxic alkaloid nicotine, that acts synergistically with anti-digestive proteinase inhibitors [2] and diterpene glycosides (DTGs) [3], whose mode of action remains largely unknown. Indirect defenses based on the emission of volatiles that render attacked plants more attractive to natural predators of the attacking herbivores represent another efficient inducible defense [4], [5], [6], [7], [8]. The fine-tuned induction of both direct and indirect defenses is thought to reduce the costs of constitutive defense protection and optimizes the ability of *N. attenuata* plants to compete with neighbors [9], [10].

Dynamic changes in a plant's defense metabolism are mediated by signal transduction pathways where jasmonic acid (JA) and its derivatives, collectively referred to as jasmonates, play major roles. This conclusion is based on a large body of studies showing that mutants or transgenic lines impaired in jasmonate biosynthesis or perception are more vulnerable to a wide range of herbivorous insects [11]. Following tissue damage, JA is synthesized *de novo* upon activation of lipases which release fatty acids from membrane lipids. Free linolenic acid is oxygenated by lipoxygenase enzymes (LOX) and then converted to 12-oxo-phytodienoic acid (OPDA) through the combined action of allene oxide synthase (AOS) and allene oxide cyclase (AOC). OPDA is subsequently transformed to JA by reduction and three cycles of β-oxidation. In addition to its central signaling function for plants' anti-herbivore defenses, jasmonic acid metabolism also coordinates several aspects of plants' development including seed germination, root elongation, flower morphogenesis or fruit ripening [12], [13], [14], [15].

JA conjugation to isoleucine (Ile) is essential for the activation of a large proportion of the defense responses controlled by the JA pathway [16] but plants also produce a diverse array of JA metabolites whose function largely remains to be explored. Methyl jasmonate (MeJA) is a fragrant compound initially isolated from the flowers of *Jasminum grandiflorum* [17]. This JA metabolite ubiquitously distributed in the plant kingdom [18] has traditionally been used, due to its stability and ease of entering plant cells to be de-esterified to release JA, as a means of examining JA-dependent gene expression in various plant families. MeJA also has been thought to play important functions in inter- and intra plant signaling [19], [20], [21], [22]. However, few studies have investigated downstream responses controlled by endogenous MeJA formation and a recent study [23] suggests that MeJA itself is probably not a signaling molecule and requires de-esterification and conjugation to Ile to induce transcriptional changes in a plant.

*Arabidopsis thaliana AtJMT* (At4G36470) – the ortholog of a floral nectary-specific gene (*NTR1*) initially cloned in *Brassia campestris* – has been shown to encode an *S-adenosyl-L-methionine:jasmonic acid O-methyltransferase* responsible for MeJA formation [19], [24]. *AtJMT* ectopic expression (35S-*jmt*) has been documented as a convincing means of increasing endogenous MeJA production [19], [25], [26], [27]: in Arabidopsis it led to 3-fold increased basal MeJA levels but did not change JA accumulation. This increase in endogenous MeJA accumulation translated into constitutively higher expression levels of several JA-responsive genes (e.g. *PDF1.2* and *VSP*) and rendered 35S-*jmt* plants more resistant than WT plants to pathogen infections, notably by the necrotroph *Botrytis cinerea* [19]. *AtJMT* ectopic expression was also associated with a significant decline in seed production in Arabidopsis [25], [26], grain yield in rice [28] and minor alterations in leaf and root morphogenesis in soybean [27]. Cipollini [25], [26] attributed the reduced seed production, as well as stalk elongation, observed in Arabidopsis 35S-*jmt* plants to an exacerbated trade-off in resource allocation during the transition from vegetative to reproductive growth that resulted from the constitutive expression of costly MeJA-inducible defenses [19], [29]. None of the aforementioned studies in 35S-*jmt* plants has interpreted the fitness consequences of MeJA production in light of the downstream alterations in JA metabolism, and hence have not considered the signaling consequences that ectopic expression of *AtJMT* may have in both defense and growth processes.

We have previously shown that increasing JA-methylation in *N. attenuata* by the ectopic expression of *AtJMT* profoundly alters JA metabolism because the methylation reaction creates a strong sink that depletes endogenous pools of free JA and of JA-Ile [30]. Here we examined the ecological consequences of these alterations by transplanting 35S-*jmt* plants into *N. attenuata's* native habitat at the Great Basin Desert in Utah and evaluated their development and susceptibility to the native herbivore community. We then performed a series of targeted and metabolomics analysis of leaves obtained from field-grown 35S-*jmt* plants to understand the alterations in herbivory-induced metabolic processes responsible for the field observations.

## Results

### *Altered JA-metabolism* in 35S-*jmt* plants increases susceptibility to *Manduca sexta*

**Figure 1:**
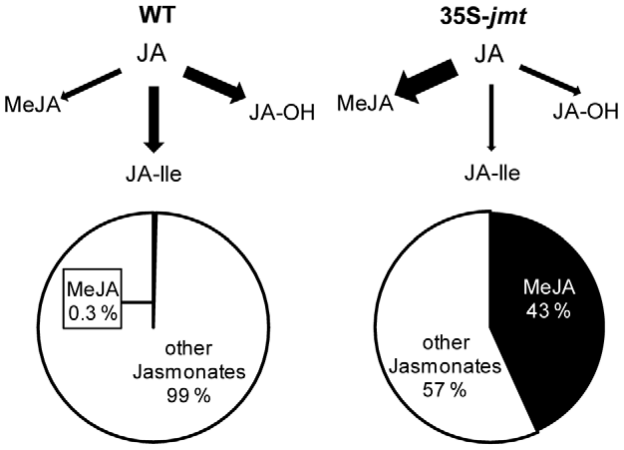
Model summarizing JA metabolism in 35S-*jmt* plants compared to in WT. Levels of MeJA and jasmonates – jasmonic acid (JA), jasmonoyl-isoleucine (JA-Ile), hydroxyl-jasmonic acid (JA-OH) – measured 30 min after elicitation by wounding and immediately applying *Manduca sexta* oral secretions to leaf puncture wounds (OS elicitation). 35S-*jmt*-1 plants are profoundly altered in JA signaling as the ectopic expression of *AtJMT* depletes free JA pools required for JA metabolism.

**Figure 2:**
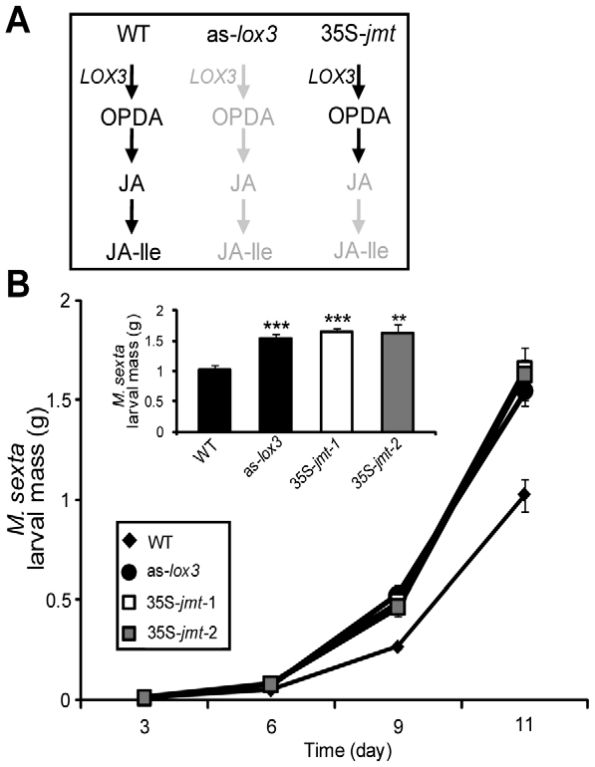
*Manduca sexta* larvae perform significantly better on 35S-*jmt* and as-*lox3* than on WT plants. (A) as-*lox3* plants, silenced in the expression of *lipoxygenase3*, whose gene-product catalyzes the first step in JA biosynthesis, have lower levels of all jasmonates. In contrast, 35S-*jmt* plants produce WT levels of all oxylipins upstream of JA. (B) Mean (± SD, n = 25) mass of *M. sexta* larvae after 3, 6, 9 and 11 days of feeding on wild-type plants (WT), *lipoxygenase3*-silenced plants (as-*lox3*), and transgenic lines ectopically expressing *AtJMT*. The bar graph represents the average larval mass at day 11. Asterisks represent significant differences among caterpillars grown on WT or transgenic lines (unpaired t-test; ** P<0.001, *** P<0.0001).

We have previously shown that ectopically expressing *AtJMT* in *N. attenuata* plants (35S-*jmt*) diverts, both in locally elicited and in systemic undamaged tissues, the flux of the jasmonate cascade towards MeJA production upon simulated *Manduca sexta* attack, and thereby reduces the pools of free JA and its metabolites [30]. This causes the insect-elicited jasmonate profiles to be dominated by MeJA in which the relative proportions of JA and its other metabolites are reduced by half compared to those found in WT (Fig. 1). This re-direction of the JA pathway reduces transcript levels of key defense-related genes that are known to be regulated by JA-signaling and the spread of elicited jasmonate accumulation patterns, all without interfering with the expression of JA biosynthesis genes. To further examine consequences on the resistance of 35S-*jmt* plants to herbivores, we compared the growth of *M. sexta* larvae feeding on two independently transformed homozygous 35S-*jmt* lines, as-*lox3* and WT plants under controlled glasshouse conditions. as-*lox3* plants are silenced for *lipoxygenase3* whose gene-product catalyzes the first step in JA biosynthesis which results in lowered levels for all jasmonates (Fig. 2A) and leads to impaired anti-herbivore defenses [31]. As previously reported [32], *M. sexta* larvae that fed on as-*lox3* plants gained significantly more mass than on WT plants (Fig. 2B). Larvae that fed on 35S-*jmt* gained as much mass as those on as-*lox3* plants, indicating that alterations in the resistance to *M. sexta* are comparably severe for the three transgenic lines (Fig. 2B): the average mass of 11-day-old caterpillars fed on 35S-*jmt*-1, 35S-*jmt*-2 and as-*lox3* lines was 61% (35S-*jmt-*1 vs WT: *P*≤0.0001), 60% (35S-*jmt*-2 vs WT: *P* = 0.0003) and 51% (as-*lox3* vs WT: *P*≤0.0001), respectively, larger than those of caterpillars fed on WT plants.

### 35S-*jmt*-1 plants do not suffer growth alterations in the field

**Figure 3:**
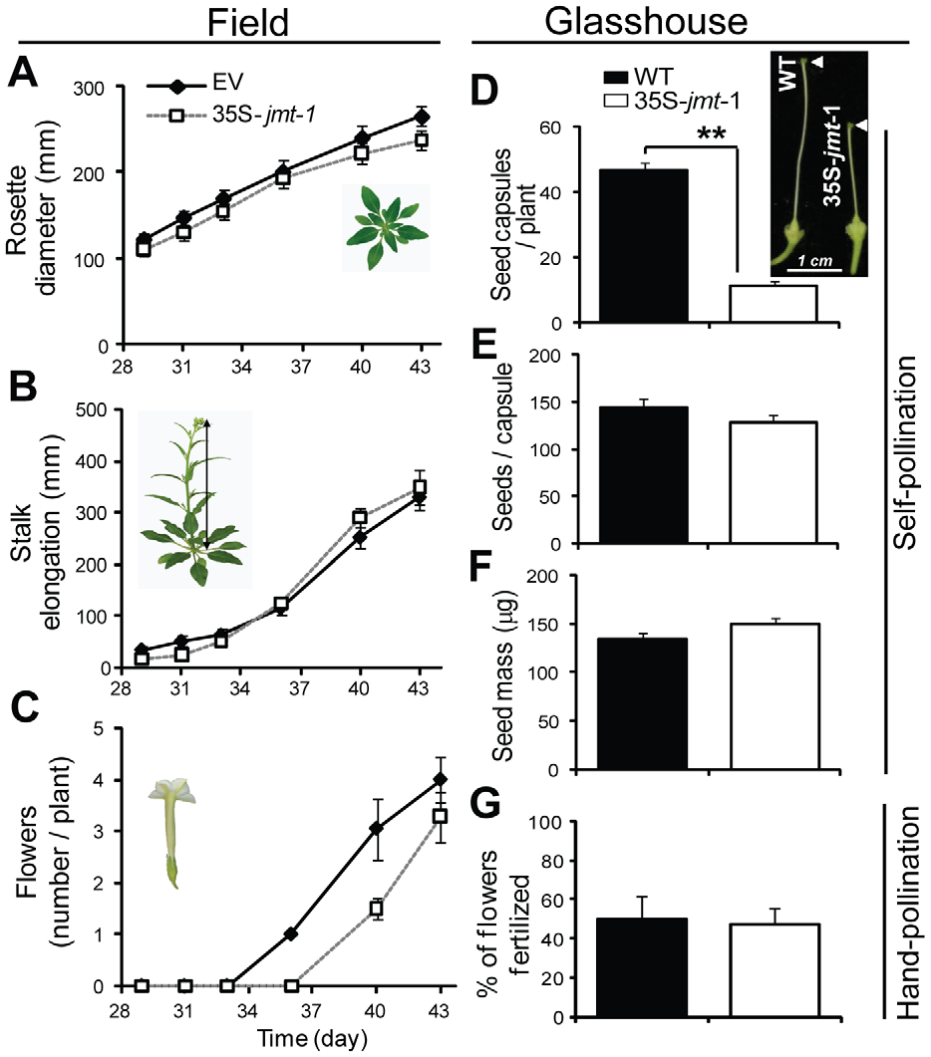
35S-*jmt*-1 plants develop as EV plants in nature but produce fewer seed capsules in the glasshouse due to impaired self-pollination. Mean (± SD, 15 to 49) rosette diameter (A) and stalk length of 35S-*jmt*-1 compared to EV plants (B). Appearance of first flowers (C) is delayed for 35S-*jmt*-1 plants. Mean capsule number (± SD, n = 6) (D), seed number per capsule (± SD, n = 25) (E) of the first four to five ripe self-pollinated capsules per plant of each genotype and seed mass (F). (G) Flowers of 35S-*jmt*-1 plants, which have reduced style length [26] (insert in panel D) produced WT numbers of seed capsules after hand-pollination with equivalent amounts of pollen. Asterisks indicate significant differences between empty-vector (EV) or WT and 35S-*jmt*-1 plants (unpaired t-test; ** P<0.001).

To determine the ecological performance of 35S-*jmt* plants, we transplanted during the 2009 field season one 35S-*jmt* line (35S-*jmt*-1) into *N. attenuata's* native environment in the Great Basin Desert (Utah, USA). In nature, *N. attenuata* plants face two major stresses: a high intra-specific competition due to their synchronized germination from seed banks after fires and a large herbivore community that reestablishes on plants emerging after fires. To simulate the intra-specific competition stress, we planted WT transformed with empty vector (EV) – to control for any transformation-related effects – and 35S-*jmt*-1 plants in pairs in close proximity. In line with earlier glasshouse-based observations [30], 35S-*jmt*-1 plants did not differ from WT or EV. The vegetative growth of 35S-*jmt*-1 plants in nature – as inferred from measures of rosette size and stalk elongation (Fig. 3A–B) – was similar to that of EV plants, indicating that 35S-*jmt*-1 did not suffer developmental consequences from competing with EV plants during vegetative growth.

### Impaired self-pollination, rather than resource allocation, reduces seed capsule production in 35S-*jmt*-1 plants

*N. attenuata* produces self-compatible flowers, which after fertilization mature into seed capsules. The appearance of the first flowers was slightly delayed in 35S-*jmt*-1 plants compared to in EV plants (Fig. 3C) but this delay did not translate into reduced flower number at later stages (data not shown). The number of seed capsules produced by 35S-*jmt*-1 plants – an important parameter to assess a plant's reproductive fitness – could not be measured in the field, as flowers had to be removed before seed capsule initiation in order to avoid the risk of disseminating genetically modified material. In the glasshouse, 35S-*jmt*-1 plants produced in average 11 while WT plants produced 46 capsules (Fig. 3D). We further counted the number of seeds per capsule and measured the average seed mass produced by each plant: no significant differences were detected (Fig. 3E–F). The reduction in seed capsule production was due to impaired self-pollination of 35S-*jmt* flowers because *N. attenuata* 35S-*jmt*-1 plants produced flowers with styles only half the length observed in WT ([30], insert in Fig. 3D). In support with impaired self-pollination being responsible for the reduced seed capsule production, the proportion of seed capsules obtained by hand-pollinated flowers was similar in 35S-*jmt*-1 and WT plants (Fig. 3G).

### 35S-*jmt*-1 plants are highly susceptible to herbivores in the field

**Figure 4:**
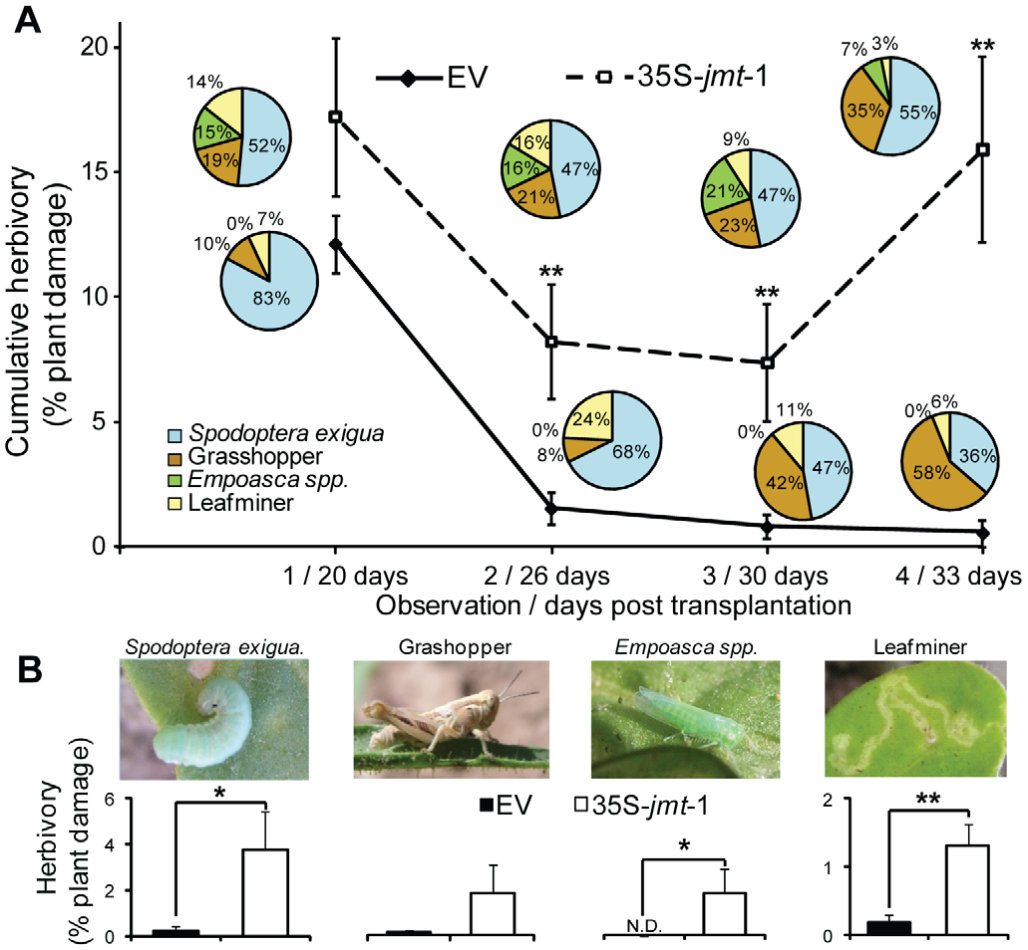
35S-*jmt*-1 plants are more vulnerable to herbivorous insects in nature. (A) Mean (± SD, n = 10 to 49) percentage of cumulative herbivory on 35S-*jmt*-1 and empty-vector (EV) transformed plants monitored 20, 26, 30 and 33 days (observations 1, 2, 3 and 4) after transplantation to the field (unpaired t-test; ** P<0.001). (B) Mean (± SD, n = 10 to 49) percentage of leaf area damaged caused by *Spodoptera exigua*, (3rd observation) grasshoppers (2nd observation), *Empoasca* spp. (3rd observation) and leaf miners (3rd observation). No significant differences were observed in the amount of damage caused by mirids (Fig. S1). Asterisks represent significant differences between EV and 35S-*jmt*-1 (unpaired t-test; * P<0.05; ** P<0.001).

The increased performance of *M. sexta* larvae on 35S-*jmt*-1 in the glasshouse (Fig. 2B), is consistent with the observation that 35S-*jmt*-1 plants were more susceptible than EV plants to herbivores of different feeding guilds in the field (Fig. 4). Although fluctuating across the different observations, cumulative foliage damage caused by Noctuidae larvae (*Spodoptera exigua*), grasshoppers (*Trimerotropis spp*.), leafhoppers (*Empoasca spp*.) and leafminers (*Diptera spp.*) was always greater on 35S-*jmt*-1 than on EV plants. Cumulative damage was statistically significantly different for the 2^nd^, 3^rd^ and 4^th^ observations (Fig. 4A; unpaired t-test, *P* = 0.0016, 0.0019 and 0.002). The relative contribution of a particular herbivore species to the total damage is displayed in pie charts for all observations. Of all types of insect damage monitored during the field season, only those caused by mirids (*Tupiocoris notatus*) did not differ significantly between 35S-*jmt*-1 and EV plants (Fig. S1). In contrast, grasshoppers showed a marginally significant feeding preference for 35S-*jmt*-1 plants and, *Spodoptera exigua* (4^th^ observation: *P* = 0.0478) and unidentified leafminers (2^nd^ observation: *P* = 0.00179) fed significantly more on 35S-*jmt*-1 plants than on EV (Fig. 4B). The characteristic leaf damage caused by attack from *Empoasca* spp. (2^nd^ observation: *P* = 0.0255) was only observed on 35S-*jmt*-1 plants (Fig. 4B).

### Herbivory-induced emissions of hexenylesters and terpenoids are reduced in 35S-*jmt*-1 plants

To infer alterations in defense metabolism responsible for the elevated vulnerability of 35S-*jmt*-1 to herbivores, we measured the production of well-established defense traits: the activity of trypsin proteinase inhibitors (TPI) [33], [34] and the accumulations of nicotine [35] and diterpene glycosides (DTGs) [3]. We additionally performed metabolomics analyses of volatile and non-volatile metabolites synthesized by leaves of field-grown plants.

**Figure 5:**
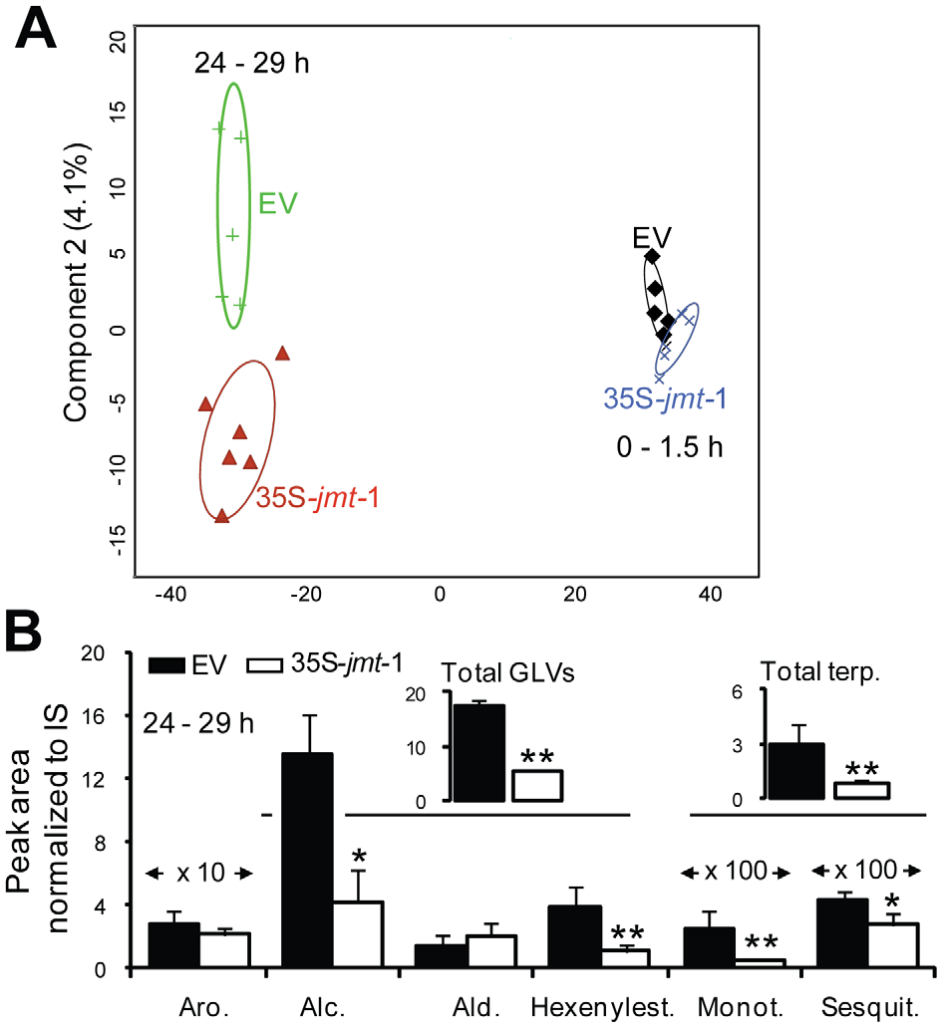
W+OS-induced emissions of hexenylesters and terpenoids by 35S-*jmt*-1 plants are reduced in field-grown plants. (A) Partial least-square discriminant analysis of 42 volatile organic compounds emitted from field-grown plants after wounding a leaf of 5 to 6 plants of each genotype with a fabric pattern wheel and applying *Manduca sexta* oral secretions (W+OS) revealed a clear distinction between volatile blends emitted by 35S-*jmt*-1 and empty-vector (EV) transformed plants 24–29 h after elicitation but not 0–1.5 h after elicitation. (B) Mean total and sub-total emissions (± SD, n = 5 to 6) for each volatiles classes emitted 24 to 29 h after W+OS elicitation. Volatile compounds are categorized as aromatic (Aro.) compounds, green leaf volatiles (GLVs) – alcohol (Alc.), aldehyde (Ald.), hexenylesters (Hexenylest.) and terpenoids (Terp.) – mono- (Mono.) and sesquiterpenes (Sesquit.). Mono- and sesquiterpenes as well as hexenyl-esters contributed the most to the genotype distinction afforded by principle component 2 (Fig. S3). Volatile emissions are expressed as peak areas standardized to the internal standard (IS) tetralin peak response. Asterisks indicate significant differences from EV plants (unpaired t-test; * P<0.05; ** P<0.001).

In nature, predators and parasitoids of herbivores have been shown to be guided to their prey by volatile organic compounds (VOCs) emitted by herbivore-damaged leaves [36], [37]. To investigate the activation of this indirect defense in field-grown 35S-*jmt*-1 plants, we collected volatiles emitted into the headspace of 35S-*jmt*-1 and EV leaves previously wounded and elicited with *M. sexta* oral secretions (W+OS). This treatment has been shown to activate both direct and indirect defenses [38], [39]. ‘Early’ volatile emissions – which mainly result from the release and oxidation of membrane lipids during the degradation of the leaf surface [40] – were collected for 1.5 h after the treatment. Volatiles emitted one photoperiod after the elicitation – here collected 24 to 29 h after W+OS – have, in contrast, been shown to be mainly under transcriptional control [38]. The 42 most abundant and consistently detected VOCs during GCxGC-TOFMS analyses of the sample-set were selected for statistical processing. ‘Early’ and ‘late’ volatile blends were clearly discriminated during both unsupervised (principal component analysis, PCA, Fig. S2) and supervised (partial least square discriminant analysis, PLSDA, Fig. 5A) clustering analysis performed on log_2_-transformed relative areas. In agreement with previous reports [41], increases in terpenoid and hexenylester production after one day in conjunction with decreased emissions of non-esterified green leaf volatiles (GLVs) accounted for this group's statistical separation in the analysis (Fig. S3).

A clear discrimination of volatile blends produced by 35S-*jmt*-1 and EV leaves was only observed 24 h after elicitation. The inspection of the loadings exerted by VOC analytes on component 2, which provided the greatest differentiation of the two genotypes, revealed that 35S-*jmt*-1 had significantly reduced emissions of *cis*-3-hexenol, hexenylesters and mono- and sesquiterpenes (Fig. 5B and Fig. S3). The largest and most significant differences (Table S1) were detected notably for the emission of *cis*-3-hexen-1-ol (83% reduction, *P* = 0.004), *cis*-3-hexenyl-butyrate (89%, *P* = 0.017) and -acetate (86%, *P* = 0.014), α-terpineol (81%, *P* = 0.002), *trans*- α -bergamotene (65%, *P* = 0.037) and ß-myrcene (20%, *P* = 0.0001). In agreement with a reduced production of certain GLVs, transcript levels for *N. attenuata hydroperoxyde lyase* (*NaHPL*) were significantly reduced in 35S-*jmt*-1 leaves compared to WT 2 h after W+OS elicitation (Fig. S4). No significant differences were observed for the emission of aromatic compounds (Fig. 5B and Table S1).

### 35S-*jmt*-1 plants failed to accumulate common defense metabolites

**Figure 6:**
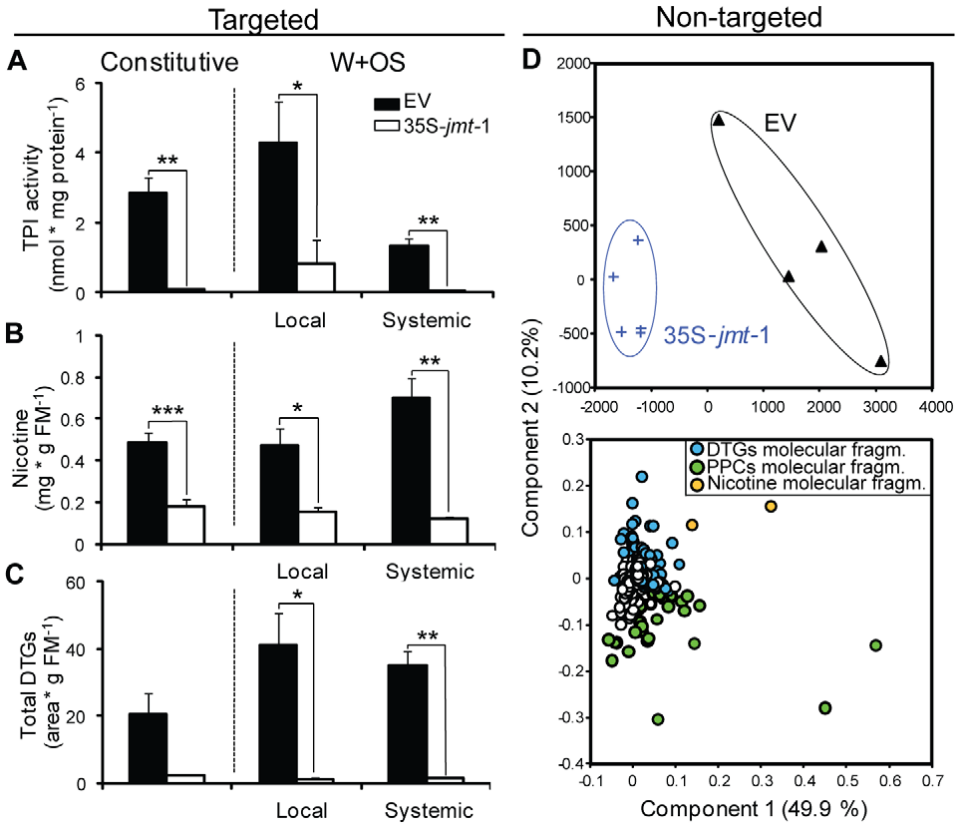
Constitutive and W+OS-induced levels of direct defenses are strongly reduced in field-grown 35S-*jmt*-1 plants. Mean (± SD, n = 5) (A) trypsin proteinase inhibitor (TPI) activity, (B) diterpene glycosides (DTGs) and (C) nicotine accumulation in rosette leaves from empty-vector (EV) transformed and 35S-*jmt*-1 plants before (‘Constitutive’) and 3 days after that one fully expanded leaf per plant was W+OS elicited. The untreated orthostichous leaf above the elicited leaf (‘Local’) was analyzed as the systemic leaf (‘Systemic’). (D) Partial least square discriminant analysis obtained after Pareto scaling revealed a clear differentiation of metabolite profiles in the W+OS-elicited leaves of 35S-*jmt*-1 and EV plants. As revealed by examining the loading plot (lower plot), ions specific for DTGs, nicotine and phenylpropanoid polyamine conjugates (PPCs) accounted for the separation between EV and 35S-*jmt*-1 profiles. The behavior of individual ions in W+OS-treated plants is summarized in File S1 and Figure S6. Molecular fragments (fragm.) correspond to discrete *m/z* features produced during the in-source ionization process. Asterisks represent significant differences between EV and 35S-*jmt*-1 plants (unpaired t-test; * P<0.05; ** P<0.001, *** P<0.0001).

Levels of TPI activity (32-fold reduction, *P* = 0.009) and nicotine accumulation (2-fold reduction, *P* = 0.0004) were significantly reduced in the leaves of field-grown 35S-*jmt*-1 plants prior W+OS induction compared to the levels in EV (‘Constitutive’, Fig. 6 A–C). Although only marginally significant (*P* = 0.052), total DTG levels were on average 10-fold reduced in 35S-*jmt*-1 leaves. Even though the eliciting effect of the W+OS treatment was not clearly apparent after 3 days – some of the leaves being obviously primed by damage inflicted by native herbivores –, levels of TPI activity, nicotine and DTGs remained strongly reduced in elicited (‘Local’) and unelicited distal leaves growing orthostichous to the elicited leaf with a minimal angle (‘Systemic’) on 35S-*jmt*-1 plants compared to EV. Local leaves harvested three days after W+OS elicitation were further analyzed using a metabolomics approach to evaluate the global impact *AtJMT* ectopic expression had on W+OS-induced secondary metabolic accumulations. ‘Local’ metabolic profiles of 35S-*jmt*-1 plants were clearly distinguished from their EV counterparts when analyzed, after Pareto-scaling, by PLSDA (Fig. 6D) indicating pronounced changes in the expression m/z signals corresponding to molecular fragments of various metabolic classes. In support with this, univariate analysis of the expression of individual *m/z* signals using SAM algorithm, a well established and highly robust statistical tool originally designed for microarray analysis [42], revealed that approximately 9% of the ions from the chromatographic profiles were more than 2-fold differentially regulated (*P*<0.05 with a false discovery rate not exceeding 1%) in 35S-*jmt*-1 leaves (Fig. S5). In addition to the above described alterations in nicotine and several DTGs, a series of phenylpropanoid-putrescine and -spermidine conjugates – compounds characterized in a previous study [43] – as well as several unknown structures (Fig. 6D, File S1), were among the metabolites which distinguished leaves of 35S-*jmt*-1 from those of EV plants. Extracted ion traces computed for the precursor *m/z* signals of different representatives of these metabolic classes are presented in Fig. S6.

### JA-Ile, but not JA complementation restores local, but not systemic, expression of certain defense traits in 35S-*jmt*-1 plants

**Figure 7:**
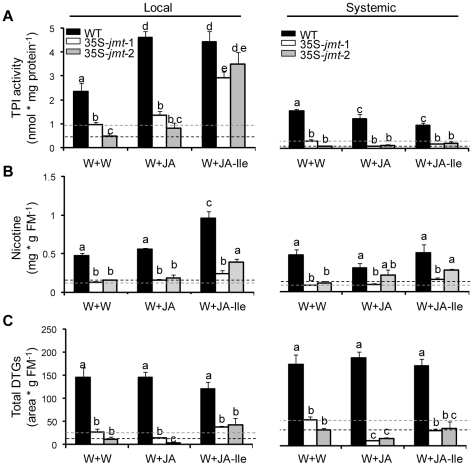
JA-Ile, but not JA complementations, restored local TPI activity but only partially nicotine and DTG production in 35S-*jmt* plants. Mean (± SD, n = 5) (A) trypsin proteinase inhibitor (TPI) activity, (B) nicotine- and (C) diterpene glycoside (DTGs) accumulation in local and systemic tissues of induced rosette-stage wild-type (WT, black bars) plants and plants ectopically expressing *AtJMT* (35S-*jmt*-1, white bars; 35S-*jmt*-2, grey bars) harvested 3 days after one fully expanded leaf per plant was wounded by a fabric pattern wheel and treated with distilled water (W+W), JA (0.1 µmoles, W+JA) or JA-Ile (0.1 µmoles, W+JA-Ile). The orthostichous leaf above the treated leaf (Local) was analyzed for the systemic response in elicited plants (Systemic). Dashed lines indicate W+W level of 35S-*jmt*-1 (grey) and 35S-*jmt*-2 (black). Bars sharing the same letters are not significantly different (unpaired t-test).

We tested the hypothesis that the impaired direct defenses of 35S-*jmt* plants resulted from decreased JA-Ile formation, as AtJMT activity has been shown to largely outcompete the activity of JAR enzymes responsible for JA-Ile synthesis in *N. attenuata* [30], by conducting complementation experiments with JA and JA-Ile. To test glasshouse-grown plants, JA and JA-Ile were applied at physiological concentrations to wounded leaves of rosette-stage 35S-*jmt* and WT plants and the regulation of TPI activity, nicotine and total DTG levels, for which we have the most knowledge, was analyzed (Fig. 7). In JA-Ile treated leaves, TPI activity was completely restored to WT levels, but no effects were observed in systemic 35S-*jmt* leaves. JA-Ile also significantly induced nicotine levels in elicited leaves of 35S-*jmt* plants but these levels did not reach those detected in similarly treated WT leaves. JA, directly methylated by AtJMT activity [30], had in contrast no inducing effect on nicotine levels compared to the wound control in 35S-*jmt* plants. Differences in total DTG levels between WT and 35S-*jmt* diminished in elicited, but not systemic leaves, when leaves of 35S-*jmt* plants were treated with JA-Ile. Again, JA had also no significant effects compared to the wound control on DTG levels. JA-Ile partly restored the production of the phenylpropanoid-polyamine conjugate caffeoylputrescine: it significantly increased caffeoylputrescine concentrations in WT leaves but only slightly increased this metabolite in elicited 35S-*jmt* leaves (Fig. S7).

## Discussion

We investigated the impact of re-routing the JA pathway and increasing MeJA production on *N. attenuata* growth and herbivore resistance in its native habitat. Although not suffering from major developmental alterations, plants were more susceptible to the native herbivore community in Utah which was associated with an impaired production of direct and indirect defense compounds. This work confirms that the homeostatic control of the flux within the JA pathway and the production of JA-Ile are of central importance for the plant's inducible defenses in nature and underlines that MeJA does not have defense signaling functions by itself.

### *AtJMT* ectopic expression does not constrain development of 35S-*jmt*-1 in nature

In the field, the vegetative growth of *N. attenuata* 35S-*jmt* plants did not differ from that of EV plants. Appearance of the first flowers was slightly delayed but not to similar extent as previously seen in 35S-*jmt* Arabidopsis plants [26]. Seed capsule production was reduced in 35S-*jmt*-1 when plants were grown under glasshouse conditions. Reduced seed capsule production in 35S-*jmt* plants could result from an exacerbated resource trade-off due to MeJA hyper-accumulation, as proposed by Cipollini et al. [25], [26], or from impaired self-pollination. Our results indicate that impaired self-pollination, caused by the reduction of floral style length ([30], and Fig. 3A, insert), was likely responsible for the observed decrease in seed capsule production. Consistent with this hypothesis, the few capsules spontaneously generated in our 35S-*jmt* plants contained as many seeds as WT controls and hand-pollination of WT and 35S-*jmt*-1 flowers yielded equal numbers of seed capsules and seeds per capsule. Moreover, no evidence could be found for constitutively elevated levels of defense traits in *N. attenuata* that might result in an energetic demand and compromise seed production; to the contrary, we found that JA-inducible defenses were substantially reduced in 35S-*jmt* plants. Alterations in floral developmental processes, some being highly plant family-specific, have been described in several mutants and transgenics in which various steps in JA signaling have been disrupted [44], [45]. Ectopic *AtJMT* expression in rice inhibits spikelet development [28] while no alterations in flower morphogenesis have been reported in 35S-*jmt* transformants of Arabidopsis [19], [25], [26], [29] and soybean [27].

### 35S-*jmt* plants phenocopy the deficiencies in direct and indirect defenses of JA-deficient plants

*AtJMT* ectopic expression reduces JA and JA-Ile levels to a comparable or greater extent than previously reported for the reductions measured in transgenic *N. attenuata* lines silenced in different steps in the JA biosynthetic pathway [30]. Under glasshouse conditions, 35S-*jmt* plants were as susceptible to larvae of *M. sexta* as as-*lox3* in which the complete octadecanoid pathway is repressed. This result rules out the possibility that upstream components of the JA pathway in *N. attenuata* function in anti-herbivore defense since the JA pathway remains intact until the formation of JA in 35S-*jmt* plants [30]. In the field, 35S-*jmt* plants were more attacked than EV by herbivores likely due to reduced accumulations of nicotine, DTGs and TPI activity levels, traits whose loss has been shown to increase herbivore loads in nature [2], [35], [46] and to be transcriptionally regulated by JA signaling [47]. These field observations were also supported by glasshouse experiments which confirmed the reduced levels for TPI, DTGs and nicotine for 35S-*jmt* plants (Fig. S8) before and after W+OS induction. The feeding and oviposition of novel herbivore species on 35S-*jmt* plants not found on WT *N. attenuata* plants, also phenocopies a trait of certain JA-deficient lines; *Empoasca spp*. leafhoppers which, have been observed to feed almost exclusively on transgenic lines deficient in JA biosynthesis and perception, were also detected on 35S-*jmt*-1 plants.

Metabolomics profiling additionally revealed profound alterations in, among others, the production of OS-elicited phenolic amine conjugates that function as defense compounds [48] and whose production requires JA signaling [47]. We also hypothesize that the attraction of natural predators of herbivores to 35S-*jmt* plants following herbivore attack or OS elicitation was reduced as a result of decreased emissions of GLVs and terpenoids that function as indirect defense. As in the activation of direct defenses, alterations in volatile production likely result from transcriptional deregulation since the largest differences revealed by PLSDA were detected for ‘late’ volatile emissions. In Arabidopsis, the same genetic transformation resulted in a constitutive up-regulation of JA inducible defense-related genes [19], [25] and increased resistance to the fungal pathogen *Botrytis cinerea* or the non-host virulent bacterium, *Pseudomonas syringae* pv tomato DC 3000 [19], [29]. Even though the two biotic interactions studied (insect attack vs fungal and pathogen infections) obviously differ, we predict that the previously discussed differences in jasmonate profiles between the two transformants may have differentially influenced resistance to the different stresses. Additionally, specificities in the signaling function of individual jasmonates also need to be considered: OPDA has been shown to function as a potent defense signal in Arabidopsis [49] and increased *AOS* expression [19] in Arabidopsis 35S-*jmt* plants may translate into higher OPDA levels protecting the plants. On the other hand, OPDA is not likely to play the same defense-related role in *N. attenuata* [50].

### MeJA itself is not a signal for both direct and indirect defenses in *N. attenuata*

Although exogenous application of MeJA has traditionally been used to analyze JA-dependent gene expression [51], [52], [53], the exact function of endogenous MeJA formation for plants' defense against herbivores and pathogens has remained largely unexplored. In contrast with previous observations in Arabidopsis 35S-*jmt* transformants [19], our study demonstrates that *N. attenuata* 35S-*jmt* plants did not benefit, but suffer from increased MeJA levels. Altogether, our results reinforce the idea that endogenous MeJA does not act directly as a defense elicitor but rather must be de-esterified and conjugated to isoleucine, as recently demonstrated in studies that manipulated levels of the MeJA esterase (MJE) [23] and the JA amino-acid-conjugating enzymes [54]. At the molecular level, docking analysis and results from yeast two-hybrid studies of the interaction of AtCOI1 and AtJAZ1 also rule out the possibility that MeJA is a ligand of the COI1 jasmonate receptor [55], [56].

### JA-Ile and likely other jasmonates elicit local and systemic defense responses

Consistent with their jasmonate profile, the defenseless phenotype of 35S-*jmt* plants resemble that of JA-Ile-deficient plants [16], [57]. JA-Ile is not targeted for methylation in 35S-*jmt* plants [30] and therefore its capacity to induce certain direct defense traits when applied to 35S-*jmt* plants reflects its signaling function. In contrast, the absence of eliciting effects of the JA treatment is consistent with the fact that JA is directly methylated and thereby diverted from JA-Ile formation, as previously observed during leaf infiltration experiments [30]. Moreover, we have shown that JA-methylation largely out-competes MJE activity and that silencing the *MJE* expression in 35S-*jmt* plants does not significantly amplify the alterations of the jasmonate profile [30].

TPI activity in local tissues of 35S-*jmt* plants was nearly restored to WT levels by JA-Ile complementation, while local DTGs and nicotine accumulations attained only half the levels detected in WT plants. These results are consistent with earlier findings of Wang et al. [57] comparing induced defenses in as-*lox3* and *JAR4/6*-silenced plants which both are JA-Ile deficient. Wang et al. showed that JA-Ile is the key signal for the local induction of TPI activity, but that the complete restoration of nicotine requires the additional action of other *LOX3*-dependent oxylipins. Our data are consistent with the conclusion that JA and/or some of its metabolites, rather than JA precursors, complement or tailor JA-Ile signaling in *N. attenuata*. JA-Ile is not translocated in *N. attenuata* [57] and conversely likely not the systemic signal for defense induction. At least three hypotheses can be formulated to explain why the induction of direct defense traits was more severely altered in systemic than in local tissues of 35S-*jmt* plants: *(i)* the local production of a JA metabolite or JA-regulated signal acting systemically is impaired in elicited leaves of 35S-*jmt* plants, *(ii)* a JA-independent systemic signal is produced locally and induces the formation of JA-Ile in systemic leaves as proposed by Koo et al. [58] (which cannot occur in 35S-*jmt* plants due to the direct methylation of JA) or *(iii)* both local and systemic processes are disrupted. Grafting experiments with WT and 35S-*jmt* plants or the use of transgenic lines expressing *AtJMT* under the control of an inducible promoter will be required to disentangle the different effects rerouting JA metabolism has in elicited and systemic tissues.

To summarize, this study reinforces the idea that the tight control exerted on the flux of compounds within the JA pathway to maximize JA-Ile production at the damage site is a central determinant of a plant's resistance to herbivores in nature. This work further highlights the value of creating metabolic sinks in the JA pathway enabling the analysis of downstream consequences in a plant's metabolism. Future work will evaluate from a metabolomics standpoint the complementation of JA-dependent responses by different jasmonates.

## Materials and Methods

### Plant material and growing conditions

Wild-type *Nicotiana attenuata* Torr. ex Watson from an inbred line in its 30^th^ generation were used for all experiments. The original collection of the seeds was done on private lands (DI ranch in southwestern Utah, USA) and since *N. attenuata* is not an endangered plant species no permit collections were necessary. Moreover, all seeds used in the study were bred in the glasshouse at our institute. Before germination on agar plates containing Gamborg B5 media, all seeds were sterilized and incubated with diluted smoke and 0.1 M GA_3_, as described previously in [59]. Plants were grown with a day/night cycle of 16 h (26°C–28°C)/8 h (22°C to 24°C) under supplemental light from Master Sun-T PIA Agro 400 or Master Sun-TPIA Plus 600-W sodium lights (Philips).

The complete cDNA sequences of *AtJMT (JASMONIC ACID O-METHYLTRANSFERASE)* and the *hptII* hygromycin resistance genes were inserted into the pRESC2 transformation vector, both as sense constructs under the control of the CaMV 35S promoter. The vector was then transformed into *N. attenuata* WT plants using *Agrobacterium tumefaciens*-mediated transformation [59]. Homozygosity of the resulting T_2_ plants was determined by screening for the resistance to the antibiotic, hygromycin. The transformation and molecular characterization of 35*S*-*jmt* plants is explained in [30].

### Insect rearing and plant treatments

Eggs of the tobacco hornworm *Manduca sexta* were obtained from North Carolina State University (Raleigh, NC, USA) and kept in a growth chamber until hatching. The freshly hatched larvae were directly placed on fully developed leaves of rosette stage plants. *M. sexta* oral secretions (OS) were collected from 3^rd^–4^th^ instar larvae reared on *N. attenuata* WT leaves as described in Roda *et al.* [60]. For all glasshouse experiments, plant treatments were randomly assigned and the first fully elongated leaf (+1 position) of rosette stage plants was treated. *M. sexta* feeding was simulated by wounding a leaf with a fabric pattern wheel on both sides of the midrib and immediately applying 20 µL of *M. sexta* OS (diluted 1∶10 in water) to fresh wounds (W+OS); this procedure which is referred to as OS-elicitation, provides a convenient means of accurately standardizing herbivore elicitation of *N. attenuata* plants. The untreated leaf growing on the same plant with a minimal angular distance above the treated leaf and therefore orthostichous to the treated leaf was considered the systemic untreated leaf. Responses inherent to the mechanical damage were monitored by applying 20 µL of de-ionized water onto the wounds (W+W). Effects of jasmonates on defense expression were tested by applying either 0.1 µM JA or JA-Ile (in 12.5% ethanol) onto wounded leaves. A 12.5% ethanol solution was applied onto wounded leaves to monitor the effect of ethanol.

### Caterpillar feeding assay

The location of rosette stage WT, 35S-*jmt* and as-*lox3* plants were randomized on the glasshouse table and one freshly hatched *M. sexta* larva was placed on the transition leaf of each plant (25 plants per line). The mass of each caterpillar was recorded after 3, 6, 9 and 11 days.

### Field experiments

All necessary permits were obtained for the described field studies. Plants transformed with an empty vector (EV) and 35S-*jmt*-1 plants (APHIS number NA146) were germinated as described above. After carefully hardened to the natural conditions (high sun/UV exposure and low relative humidity) for 1 week in a mesh tent, plants were transplanted in a randomized manner to a field plot on the Lytle Preserve research station (Utah, USA) providing a native habitat. Losses to the plants' canopy caused by the native community of herbivores were measured as the total plant area damaged. Cumulative insect damage was monitored 4 times: 20, 26, 30 and 33 days after plants were transplanted to the field. Damages specifically inflicted by *Spodoptera exigua* larvae, grasshoppers, *Empoasca* spp., leafminers (*Diptera spp.*) and mirids (*Tupiocoris notatus*) were identified according to Pandey et. al [61] and recorded separately. Due to the massive damage inflicted to certain plants and the use of others for further experiments, replicate numbers varied across the different observations: EV, observations 1–3 n = 49; observation 4: n = 35; 35S-*jmt*-1, n = 24; observation 4: n = 10.

### TPI activity measurement

TPI activity of each five biological replicates was analyzed using the radial diffusion activity assay described in van Dam *et al.* [33].

### Volatile collection and analysis

In the field, one fully expanded rosette leave was OS-elicited (W+OS) and enclosed in food-quality plastic chambers with holes at both ends. On one end, the air was pulled through the chamber and on the other end trapped with a pump equipped with a charcoal trap (Orbo M32, Sigma-Aldrich, Germany) as described in Kessler and Baldwin [31]. Volatile blends of each of the six biological replicates were trapped from 0 to 1.5 and 24 to 29 hours after elicitation. Charcoal traps (ORBO-32 SMALL 100/50MG PK/50, Product Number 20267-U, Sigma-Aldrich, Germany) were stored at −20°C until use. In the lab, charcoal traps were spiked with 400 ng of tetralin (Sigma-Aldrich, Germany) used as internal standard and then eluted with 500 µL dichloromethane into a GC-vial equipped with a glass insert. All samples were analyzed on a Agilent 6890N gas chromatograph (Agilent Technologies, Böblingen, Germany) coupled with a LECO Pegasus III time-of-flight mass spectrometer with a 4D thermal modulator upgrade (LECO, Mönchengladbach, Germany) and deconvoluted as described by Gaquerel *et al.* [43]. Processed peaks were reported at a signal to noise ratio of 10 and their identification was performed against home-made libraries of authentic standards. Areas of target analytes were reported relative to the tetralin peak area. Principal component (PCA) and partial least square discriminant (PLSDA) analyses were performed on log_2_-transformed data using the Metaboanalyst web server (http://www.metaboanalyst.ca/).

### Targeted analysis of nicotine and diterpene glycosides

Nicotine and diterpene glycosides (DTGs) were extracted from five biological replicates and analyzed by high-performance liquid chromatography (HPLC) as previously described [32] with the following modifications to the extraction procedure: approximately 100 mg of frozen tissue was homogenized in 2 mL reaction tubes containing two 5 mm steel beads with a Genogrinder© by shaking at a frequency of 1200 strokes/minute for 5 min in 1 mL of 40% methanol and 0.5% acetic acid in de-ionized water as extraction buffer.

### Non-targeted leaf metabolite profiling

Two microliters of the 40% methanol leaf extract used for the targeted nicotine and diterpene glycosides analysis were separated using a Dionex rapid separation liquid chromatography system (RSLC) system (Dionex, Sunnyvale, USA). The column used was a 150×2 mm, Phenomenex Gemini NX 3u, column (150×2 mm) with a 4 mm×4 mm i.d. guard column of the same material. The following binary gradient was applied: 0 to 0.5 min, isocratic 80% A (de-ionized water, 0.1% [v/v] acetonitrile and 0.05% formic acid), 20% B (acetonitrile and 0.05% formic acid); 0.5 to 2 min, gradient phase to reach 60% A, 40% B; 2 to 6 min, isocratic 60% A, 40% B and 6 to 10 min, gradient phase to reach 20% A, 80% B. Flow rate was 200 µL/min. Eluted compounds were detected by a MicroToF mass spectrometer (Bruker Daltonics, Bremen, Germany) equipped with an electrospray ionization source in positive ionization mode. Typical instrument settings were as follows: capillary voltage 4500 V, capillary exit 130 V, dry gas temperature 200°C, dry gas flow of 8 L/min. Ions were detected from *m/z* 200 to 1400 at a repetition rate of 1 Hz. Mass calibration was performed using sodium formate clusters (10 mM solution of NaOH in 50/50% v/v isopropanol/water containing 0.2% formic acid).

Raw data files were converted to netCDF format using the export function of the Data Analysis v4.0 software (Bruker Daltonics, Bremen, Germany). Peak detection was performed using the ‘centWave’ method [62] of the R package XCMS and the parameter settings ppm = 20, snthresh = 4, peakwidth = 5–18 s. Retention time correction was achieved using the parameter settings minfrac = 0.1, bw = 10 s, mzwid = 0.05 Da. Ion traces were deconvoluted and putative in-source pseudo-spectra reconstructed with the R package CAMERA with defaults parameters. Isotopic peaks and multi-charged *m/z* signals detected by CAMERA were excluded to reduce the redundancy within the data matrix. Consistent mass features – present (for a single plant genotype and tissue) in four out of the five biological replicates – with a retention time >50 s were considered for statistical analysis. Differential expression in *m/z* feature intensity was assessed using the SAM algorithm implemented in TIGR MEV 4.6 (http://www.tm4.org/mev/). *P* values (two-class unpaired comparison) were obtained from permutation tests (1000 permutations). SAM gives estimates of the False Discovery Rate (FDR), which is the proportion of features likely to have been identified by chance as being significant. The delta value (Delta Table, File S1) was set in order than the FDR did not exceed 1%. The fold-change (35S-*jmt* W+OS-elicited>EV W+OS-elicited) threshold was set to 2. PLSDA was performed as described the “Volatile collection and analysis” paragraph.

## Supporting Information

Figure S1 **35S-** ***jmt*** **-1 plants were as vulnerable to herbivorous mirids as EV plants in the field.** Mean (± SD) damage caused by mirids (*Tupiocoris notatus*) to field grown 35S-*jmt*-1 and empty-vector (EV) plants measured 20 days after plants were transplanted to the field. Asterisks represent significant differences between EV and 35S-*jmt*-1 (unpaired t-test; * P<0.05).
(TIF)

Figure S2 **Principal component analysis of volatile blends emitted by 35S-** ***jmt*** **-1 and EV plants in the field.** Principal component (PC) analysis of 42 volatile organic compounds emitted from field-grown plants after wounding and OS-elicitation of leaf (W+OS) revealed a distinction between volatile blends emitted by 35S-*jmt*-1 and empty-vector (EV) transformed plants 24–29 h after elicitation but not 0–1.5 h after elicitation. Volatile emissions were expressed as peak areas standardized to the internal standard (IS) tetralin peak response and log_2_-transformed before analysis.
(TIF)

Figure S3 **Loadings exerted on component 1 and component 2 of the PLSDA by each of the 42 most abundant volatiles emitted in nature.** The 42 most abundant and consistently detected VOCs during GCxGC-TOFMS analyses of the sample-set were selected for statistical processing. Volatile compounds are categorized as aromatic (Aro.) compounds, green leaf volatiles (GLVs) – alcohol (Alc.), aldehyde (Ald.), hexenylesters (Hexenylest.) and terpenoids (Terp.) – mono- (Mono.) and sesquiterpenes (Sesquit.). Mono- and sesquiterpenes as well as hexenyl-esters contributed the most to the genotype distinction afforded by component 2 (Figure 5A). ‘Early’ and ‘late’ volatile blends were clearly discriminated on the first component of the partial least square discriminant analysis, PLSDA (Figure 5A) projection plot. In agreement with previous reports [36] and as visualized from the examination of the loadings on component 1, increases in terpenoid and hexenylester production after one day concomitant with vanishing emissions of non-esterified GLVs accounted for this group separation. Volatile emissions are expressed as peak areas standardized to the internal standard (IS) tetralin peak response and log_2_-transformed before analysis.
(TIF)

Figure S4 **Ectopically expressing AtJMT in** ***N. attenuata*** **reduces herbivory-induced transcript levels of Na** ***HPL*** **.** Mean (± SD, n = 5) accumulation of *hydroperoxide lyase* (*HPL*) transcripts 2 h after W+OS elicitation. Asterisks represent significant differences between WT and 35S-*jmt*-1 (unpaired t-test; *** P<0.0001). NaHPL transcripts were quantified as described in File S2.
(TIF)

Figure S5 **Differential regulation of the metabolomic profile of field grown 35S-** ***jmt*** **-1 plants after W+OS elicitation compared to EV controls.** Differential expression in m/z features of the metabolomic profiles of field grown 35S-*jmt*-1 and EV control plants was assessed using the SAM algorithm implemented in the TIGR MEV platform version 4.6. The delta value (Delta Table in File S1) was set in order than the FDR did not exceed 1%. The fold-change threshold was set to 2.
(TIF)

Figure S6 **Representative (n = 4–5) UPLC-TOFMS extracted ion chromatograms computed for precursor ions corresponding to different metabolic classes deregulated in 35S-** ***jmt*** **field-grown plants.** Elemental formulas (Elem. Formula) – relative mass errors (in ppm) were for all predicted elemental formulas below 8 ppm – were calculated using Smart Formula from the UPLC-TOFMS operating software. Candidate formulas were ranked according to both mass deviation and isotope pattern accuracy reflected in the sigma value. MS/MS+ spectra for some of the reported parent ion have been published by our group in [39] and the strategy used for compound annotation is explained in this publication. Indexes after nitrogen atoms indicate that structural rearrangements during in-source or CID-MS/MS fragmentation did not allow the unequivocal assignment of the phenylpropanoid residues to either the N1, N5, or N10 positions of spermidine. C: core molecule; G: glycosylated DTG; G+M: glycosylated+malonylated.
(TIF)

Figure S7 **Caffeoylputrescine is absent from extracts of 35S-** ***jmt*** **-1 plants and partly restored in JA-Ile, but not in JA complemented leaves.** Mean (± SD) caffeoylputrescine accumulation in local and systemic leaves of induced rosette-stage leaves from wild-type (WT, black bars) and lines ectopically expressing *AtJMT* (35S-*jmt*-1, white bars; 35S-*jmt*-2, grey bars) harvested 3 days after one fully expanded leaf per plant was wounded by a fabric pattern wheel and treated with distilled water (W+W), JA (0.1 µmoles, W+JA) or JA-Ile (0.1 µmoles, W+JA-Ile). Bars sharing the same letters are not significantly different (unpaired t-test, n = 5).
(TIF)

Figure S8 **Constitutive and W+OS-induced levels of direct defenses are strongly reduced in glasshouse grown 35S-** ***jmt*** **-1 plants.** Mean (± SD, n = 5) (A) trypsin proteinase inhibitor (TPI) activity, (B) nicotine and (C) diterpene glycosides (DTGs) accumulation in rosette leaves from wild type (WT) and 35S-*jmt*-1 plants before (‘Constitutive’) and 3 days after that one fully expanded leaf per plant was W+OS elicited. The untreated orthostichous leaf above the elicited leaf (‘Local’) was analyzed as the systemic leaf (‘Systemic’). Asterisks represent significant differences between WT and 35S-*jmt*-1 plants (unpaired t-test; * P<0.05; ** P<0.001, *** P<0.0001).
(TIF)

File S1 **Differentially regulated and annotated** ***m/z*** **features revealed by metabolomics analysis of field-grown 35S-** ***jmt*** **-1 plants.**
(XLS)

File S2 **Methods for Figure S4.**
(DOCX)

Table S1 **W+OS-elicited emissions of the 20 most abundant VOCs.** Mean (± SD, n = 5 to 6) emissions of terpenoids, GLVs and aromatic compounds of empty-vector (EV) and 35S-*jmt*-1 plants relative to the internal standard, tetralin. One leaf (+1 position) of each rosette stage plant was mechanically wounded and treated with *M. sexta* oral secretions (W+OS). Volatiles were collected 24 to 29 h after elicitation. Samples were analyzed by GCxGC-TOF-MS and two-dimensional separations were attained using an RTX-5MS column followed by a DB-17 column, providing retention time *RT1* (first dimension) and *RT2* (second dimension) as described in Gaquerel *et al.* [41]. Asterisks represent significant differences between WT and 35S-*jmt*-1 (unpaired t-test; * P<0.05; ** P<0.001, *** P<0.0001; n.d. = not detected).
(DOCX)
