# Supplementary figures and images for: Diverting the Flux of the JA Pathway in Nicotiana attenuata Compromises the Plant's Defense Metabolism and Fitness in Nature and Glasshouse
Source: PLoS One. 2011 Oct 10;6(10):e25925. doi: 10.1371/journal.pone.0025925 (PMC3189938; doi:10.1371/journal.pone.0025925)

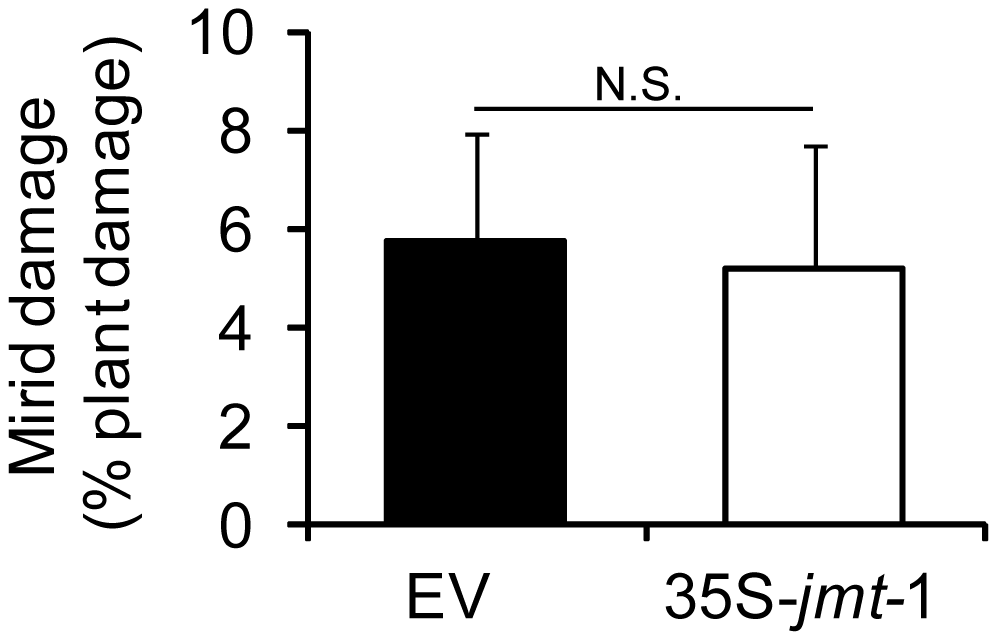

Supplement: Figure S1 — 35S- jmt -1 plants were as vulnerable to herbivorous mirids as EV plants in the field. Mean (± SD) damage caused by mirids (Tupiocoris notatus) to field grown 35S-jmt-1 and empty-vector (EV) plants measured 20 days after plants were transplanted to the field. Asterisks represent significant differences between EV and 35S-jmt-1 (unpaired t-test; * P<0.05). (TIF) [file pone.0025925.s001.tif]

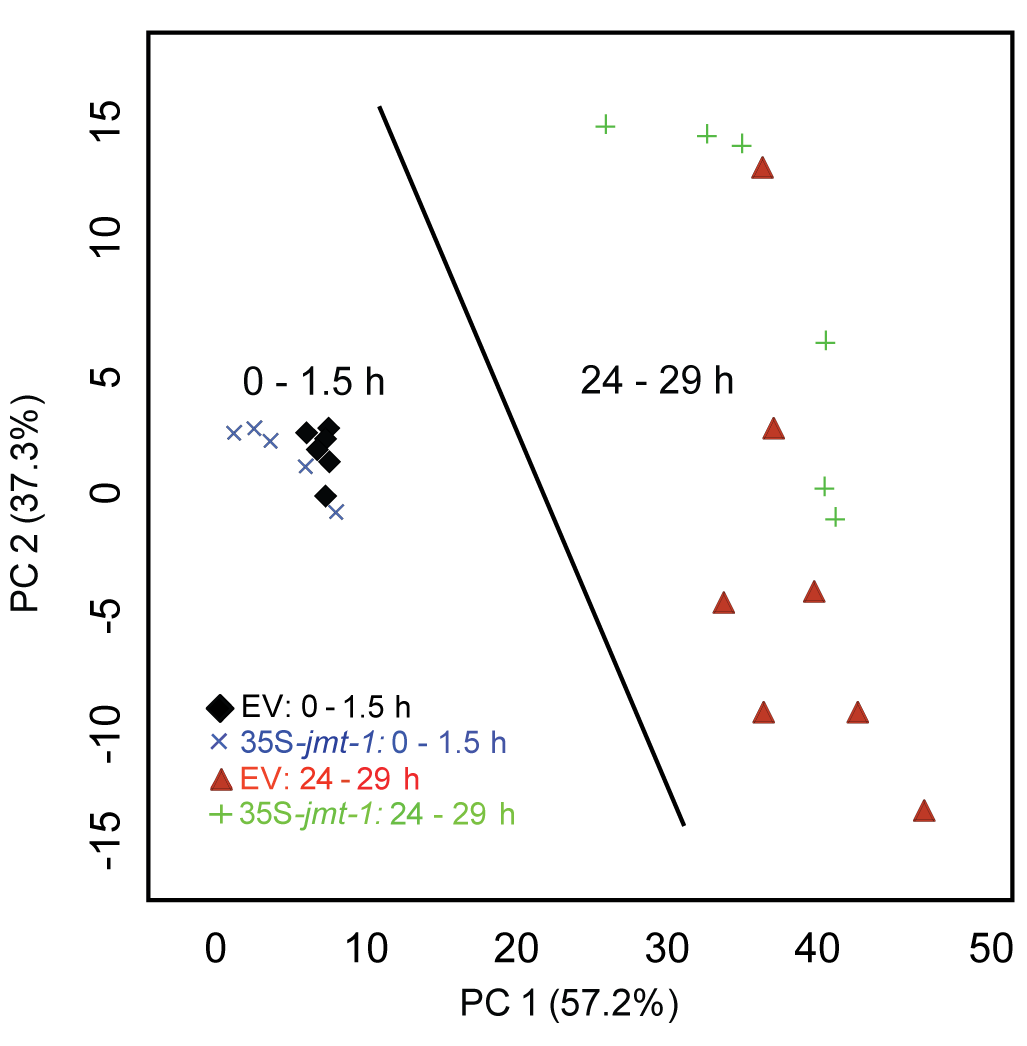

Supplement: Figure S2 — Principal component analysis of volatile blends emitted by 35S- jmt -1 and EV plants in the field. Principal component (PC) analysis of 42 volatile organic compounds emitted from field-grown plants after wounding and OS-elicitation of leaf (W+OS) revealed a distinction between volatile blends emitted by 35S-jmt-1 and empty-vector (EV) transformed plants 24–29 h after elicitation but not 0–1.5 h after elicitation. Volatile emissions were expressed as peak areas standardized to the internal standard (IS) tetralin peak response and log2-transformed before analysis. (TIF) [file pone.0025925.s002.tif]

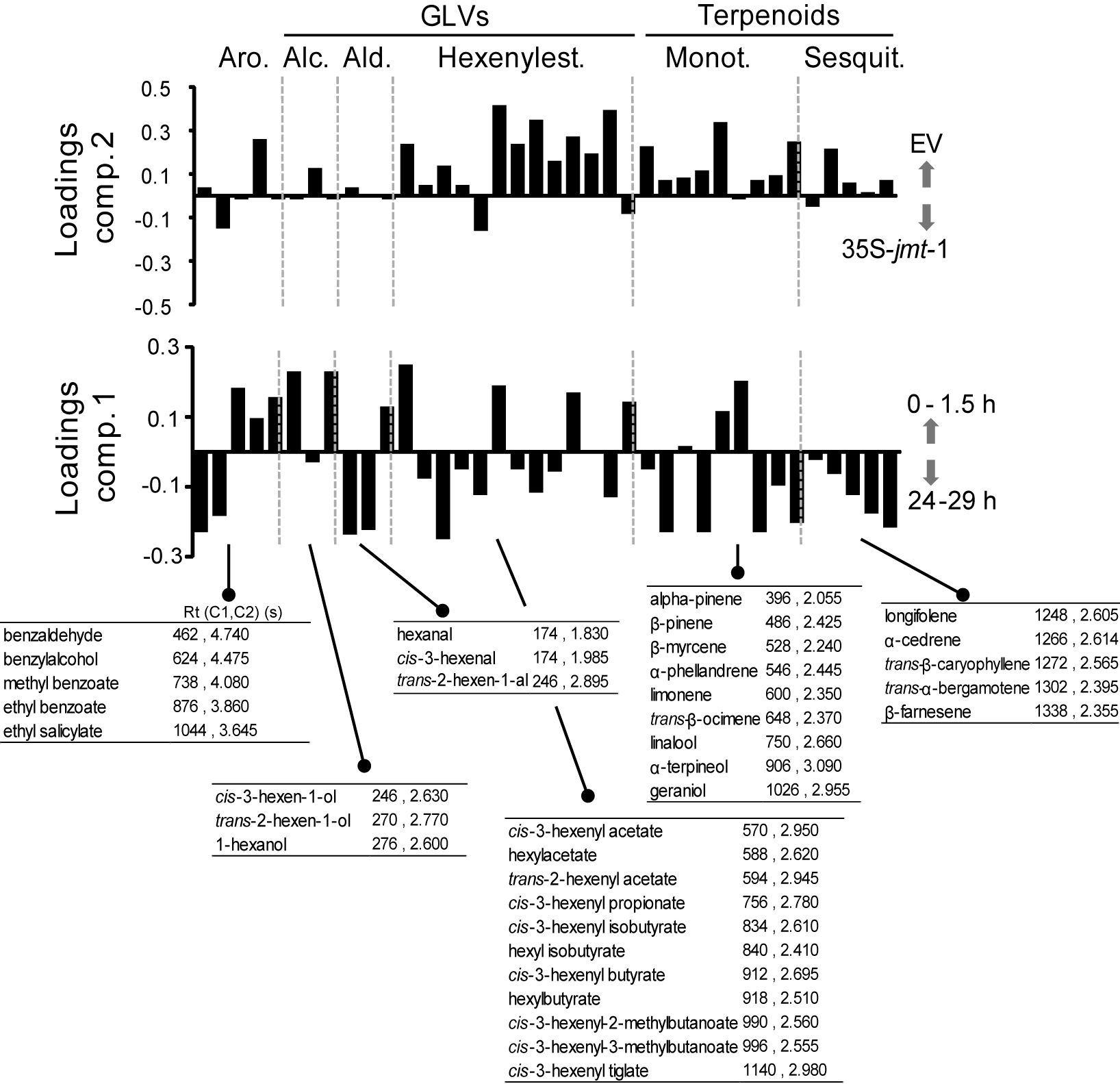

Supplement: Figure S3 — Loadings exerted on component 1 and component 2 of the PLSDA by each of the 42 most abundant volatiles emitted in nature. The 42 most abundant and consistently detected VOCs during GCxGC-TOFMS analyses of the sample-set were selected for statistical processing. Volatile compounds are categorized as aromatic (Aro.) compounds, green leaf volatiles (GLVs) – alcohol (Alc.), aldehyde (Ald.), hexenylesters (Hexenylest.) and terpenoids (Terp.) – mono- (Mono.) and sesquiterpenes (Sesquit.). Mono- and sesquiterpenes as well as hexenyl-esters contributed the most to the genotype distinction afforded by component 2 (Figure 5A). ‘Early’ and ‘late’ volatile blends were clearly discriminated on the first component of the partial least square discriminant analysis, PLSDA (Figure 5A) projection plot. In agreement with previous reports [36] and as visualized from the examination of the loadings on component 1, increases in terpenoid and hexenylester production after one day concomitant with vanishing emissions of non-esterified GLVs accounted for this group separation. Volatile emissions are expressed as peak areas standardized to the internal standard (IS) tetralin peak response and log2-transformed before analysis. (TIF) [file pone.0025925.s003.tif]

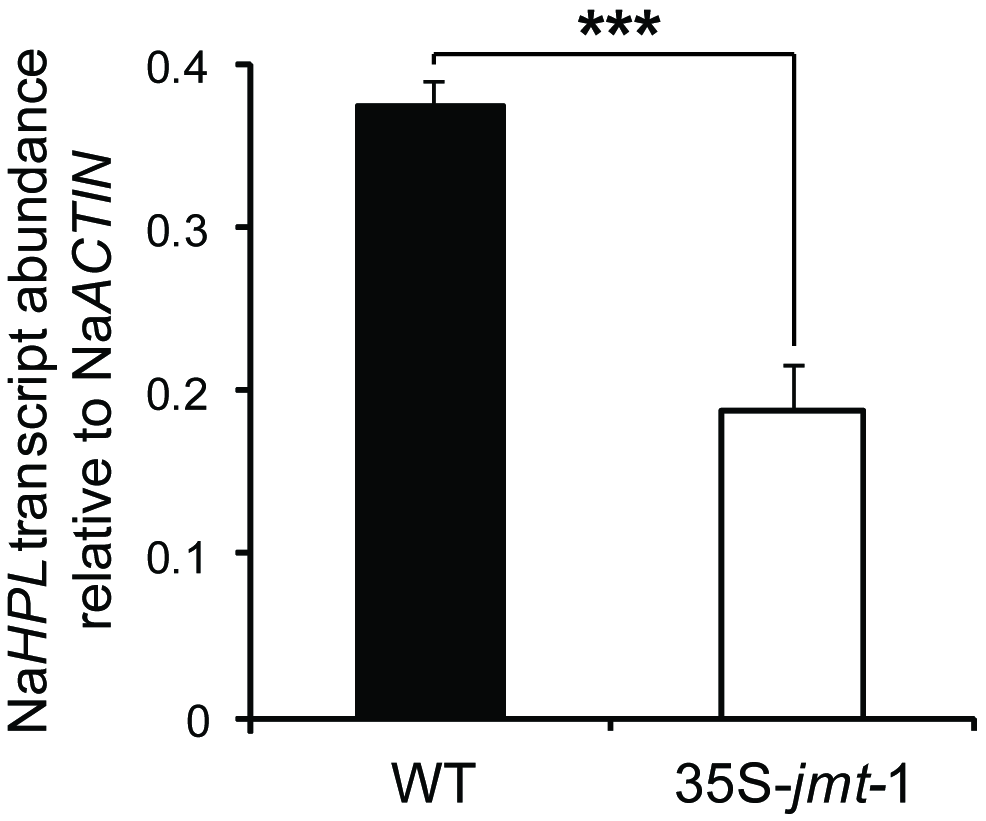

Supplement: Figure S4 — Ectopically expressing AtJMT in N. attenuata reduces herbivory-induced transcript levels of Na HPL . Mean (± SD, n = 5) accumulation of hydroperoxide lyase (HPL) transcripts 2 h after W+OS elicitation. Asterisks represent significant differences between WT and 35S-jmt-1 (unpaired t-test; *** P<0.0001). NaHPL transcripts were quantified as described in File S2. (TIF) [file pone.0025925.s004.tif]

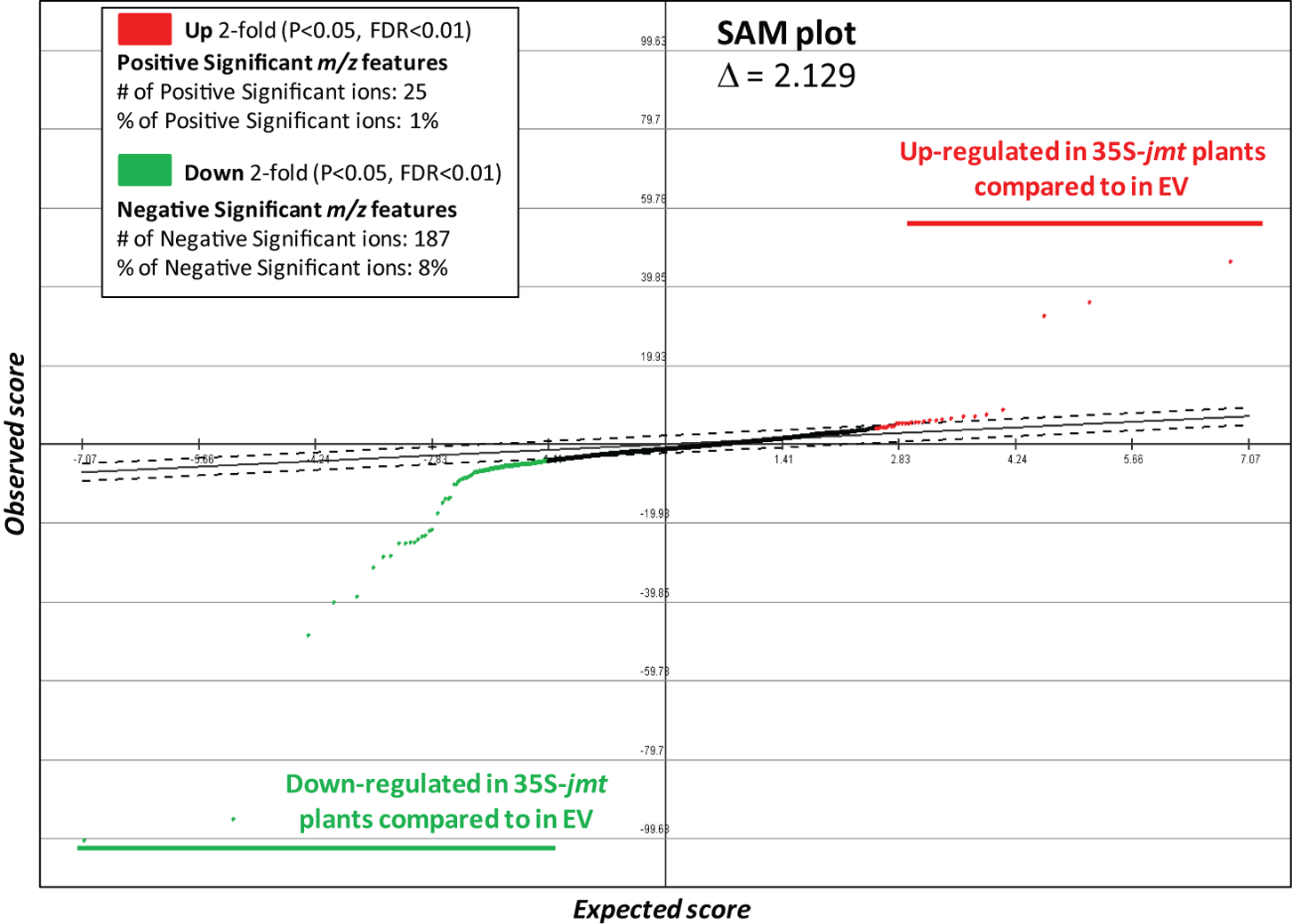

Supplement: Figure S5 — Differential regulation of the metabolomic profile of field grown 35S- jmt -1 plants after W+OS elicitation compared to EV controls. Differential expression in m/z features of the metabolomic profiles of field grown 35S-jmt-1 and EV control plants was assessed using the SAM algorithm implemented in the TIGR MEV platform version 4.6. The delta value (Delta Table in File S1) was set in order than the FDR did not exceed 1%. The fold-change threshold was set to 2. (TIF) [file pone.0025925.s005.tif]

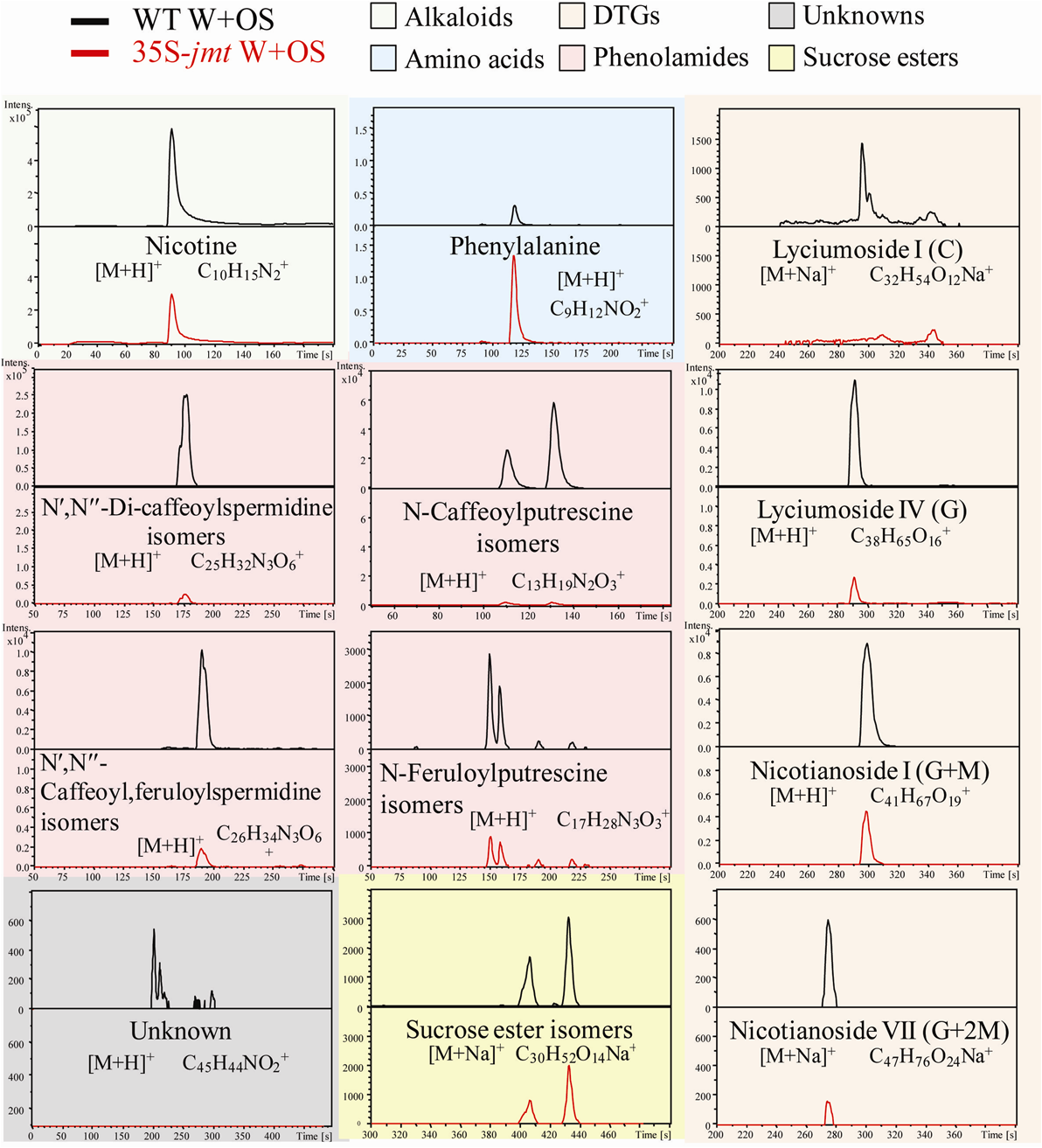

Supplement: Figure S6 — Representative (n = 4–5) UPLC-TOFMS extracted ion chromatograms computed for precursor ions corresponding to different metabolic classes deregulated in 35S- jmt field-grown plants. Elemental formulas (Elem. Formula) – relative mass errors (in ppm) were for all predicted elemental formulas below 8 ppm – were calculated using Smart Formula from the UPLC-TOFMS operating software. Candidate formulas were ranked according to both mass deviation and isotope pattern accuracy reflected in the sigma value. MS/MS+ spectra for some of the reported parent ion have been published by our group in [39] and the strategy used for compound annotation is explained in this publication. Indexes after nitrogen atoms indicate that structural rearrangements during in-source or CID-MS/MS fragmentation did not allow the unequivocal assignment of the phenylpropanoid residues to either the N1, N5, or N10 positions of spermidine. C: core molecule; G: glycosylated DTG; G+M: glycosylated+malonylated. (TIF) [file pone.0025925.s006.tif]

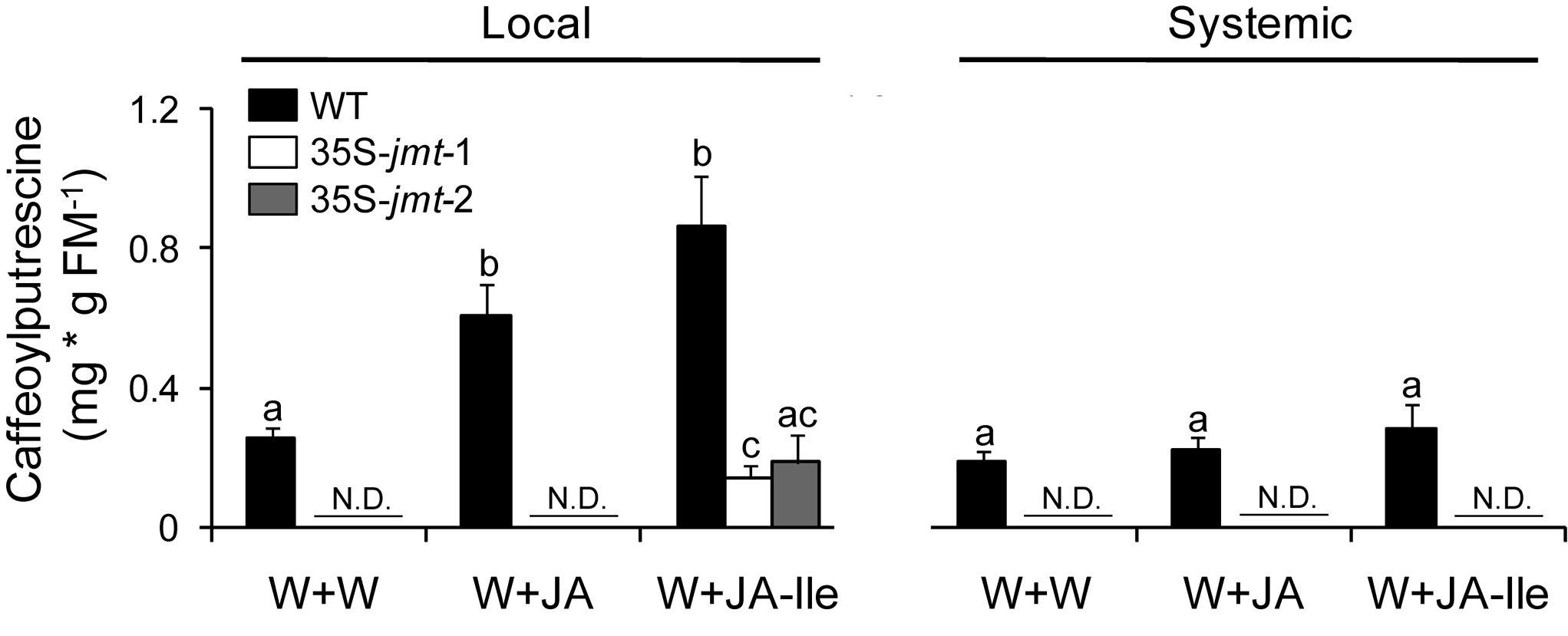

Supplement: Figure S7 — Caffeoylputrescine is absent from extracts of 35S- jmt -1 plants and partly restored in JA-Ile, but not in JA complemented leaves. Mean (± SD) caffeoylputrescine accumulation in local and systemic leaves of induced rosette-stage leaves from wild-type (WT, black bars) and lines ectopically expressing AtJMT (35S-jmt-1, white bars; 35S-jmt-2, grey bars) harvested 3 days after one fully expanded leaf per plant was wounded by a fabric pattern wheel and treated with distilled water (W+W), JA (0.1 µmoles, W+JA) or JA-Ile (0.1 µmoles, W+JA-Ile). Bars sharing the same letters are not significantly different (unpaired t-test, n = 5). (TIF) [file pone.0025925.s007.tif]

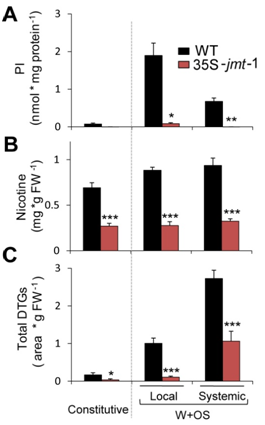

Supplement: Figure S8 — Constitutive and W+OS-induced levels of direct defenses are strongly reduced in glasshouse grown 35S- jmt -1 plants. Mean (± SD, n = 5) (A) trypsin proteinase inhibitor (TPI) activity, (B) nicotine and (C) diterpene glycosides (DTGs) accumulation in rosette leaves from wild type (WT) and 35S-jmt-1 plants before (‘Constitutive’) and 3 days after that one fully expanded leaf per plant was W+OS elicited. The untreated orthostichous leaf above the elicited leaf (‘Local’) was analyzed as the systemic leaf (‘Systemic’). Asterisks represent significant differences between WT and 35S-jmt-1 plants (unpaired t-test; * P<0.05; ** P<0.001, *** P<0.0001). (TIF) [file pone.0025925.s008.tif]
