# Supplementary material for: Diverting the Flux of the JA Pathway in Nicotiana attenuata Compromises the Plant's Defense Metabolism and Fitness in Nature and Glasshouse
Source: PLoS One. 2011 Oct 10;6(10):e25925. doi: 10.1371/journal.pone.0025925 (PMC3189938; doi:10.1371/journal.pone.0025925)
Supplement: File S2 — Methods for Figure S4. (DOCX) [file pone.0025925.s010.docx]

**Supplemental File S2**

**METHODS FOR FIGURE S4**

**Quantitative real-time PCR analysis**

Total RNA from five biological replicates per line was extracted as described in Linke *et al*. [1]. RNA extracts were treated with DNAse using the DNA-free™ Kit from Ambion (Applied Biosystems/Ambion, Austin). cDNA was synthesized from 500 ng RNA using SuperScript II Reverse Transcriptase (Invitrogen, Germany) and a poly-T primer. Quantitative real-time PCR (qRT-PCR, Stratagene 500 Mx3005P, Waldbronn, Germany) was conducted with 30 ng cDNA using the core reagent kit (Eurogentec, <http://www.eurogentec.be)> and pairs of gene specific primers:

*NaACTIN* forward: 5´-GGTCGTACCACCGGTATTGTG-3´

*NaACTIN* reverse: 5´-GTCAAGACGGAGAATGGCATG-3´

*NaHPL* forward: 5´-CACTTAGACTTAGTCCACCTGTGC-3´

*NaHPL* reverse: 5´-AACACAAACTTTTCAGGATCATCA-3´

qPCR products were detected after reaction with SYBR Green (qPCR Core Kit for SYBR Green I; Eurogentec, Köln, Germany). Relative gene expression was calculated using a 200-fold dilution series of cDNAs synthesized from RNA samples of the same experiment and normalized, according to Pfaffl *et al*. [2], by the expression value *N. attenuata ACTIN* gene**.**

**References:**

1. Linke C, Conrath U, Jeblick W, Betsche T, Mahn A, et al. (2002) Inhibition of the plastidic ATP/ADP transporter protein primes potato tubers for augmented elicitation of defense responses and enhances their resistance against *Erwinia carotovora*. Plant Physiology 129: 1607-1615.

2. Pfaffl MW, Horgan GW, Dempfle L (2002) Relative expression software tool (REST (c)) for group-wise comparison and statistical analysis of relative expression results in real-time PCR. Nucleic Acids Research 30.

3. Kessler A, Baldwin IT (2001) Defensive function of herbivore-induced plant volatile emissions in nature. Science 291: 2141-2144.

4. Gaquerel E, Weinhold A, Baldwin IT (2009) Molecular interactions between the specialist herbivore *Manduca sexta* (Lepidoptera, Sphigidae) and its natural host *Nicotiana attenuata*. VIII. An unbiased GCxGC-ToFMS analysis of the plant´s elicited volatile emissions. Plant Physiology 149: 1408 - 1423.
