# Supplementary material for: Diverting the Flux of the JA Pathway in Nicotiana attenuata Compromises the Plant's Defense Metabolism and Fitness in Nature and Glasshouse
Source: PLoS One. 2011 Oct 10;6(10):e25925. doi: 10.1371/journal.pone.0025925 (PMC3189938; doi:10.1371/journal.pone.0025925)
Supplement: Table S1 — W+OS-elicited emissions of the 20 most abundant VOCs. Mean (± SD, n = 5 to 6) emissions of terpenoids, GLVs and aromatic compounds of empty-vector (EV) and 35S-jmt-1 plants relative to the internal standard, tetralin. One leaf (+1 position) of each rosette stage plant was mechanically wounded and treated with M. sexta oral secretions (W+OS). Volatiles were collected 24 to 29 h after elicitation. Samples were analyzed by GCxGC-TOF-MS and two-dimensional separations were attained using an RTX-5MS column followed by a DB-17 column, providing retention time RT1 (first dimension) and RT2 (second dimension) as described in Gaquerel et al. [41]. Asterisks represent significant differences between WT and 35S-jmt-1 (unpaired t-test; * P<0.05; ** P<0.001, *** P<0.0001; n.d. = not detected). (DOCX) [file pone.0025925.s011.docx]

**Table SI. W+OS-elicited emissions for the 22 most abundant VOCs.**

Mean (± SD, n = 5 to 6) emissions of terpenoids, GLVs and aromatic compounds of Empty-Vector (EV) and 35S*-jmt1*plants relative to the internal standard, tetralin. One leaf (+1 position) of each rosette stage plant was mechanically wounded and treated with *M. sexta* oral secretions (W+OS). Volatiles were collected as described in Kessler *et al.* [1] 24 to 29 h after elicitation. Samples were analyzed by GCxGC-TOF-MS and two-dimensional separations were attained using an RTX-5MS column followed by a DB-17 column, providing retention time *RT1* (first dimension) and *RT2* (second dimension) as described in Gaquerel *et al.* [2]. Asterisks represent significant differences between WT and 35S*-jmt* -1 (unpaired t-test; * P < 0.05; ** P < 0.001, *** P < 0.0001; n.d. = not detected)

|  |  | | | | |  |  | | |  | |  |  |  |  |  |  |
| --- | --- | --- | --- | --- | --- | --- | --- | --- | --- | --- | --- | --- | --- | --- | --- | --- | --- |
| **Class** | **Compound** | **RT (s)** | |  | | | | | | | | | | | | |  |
|  |  | ***RT1*** | ***RT2*** | **EV** | | | | **35S-*jmt-*1** | | | | |  |  |  |  |  |
| **Terpenoids** | α-cedrene | 1266 | 2.61 | 0.27 | ± | 0.03 | | 0.13 | ± | | 0.01 | |  |  |  |  |  |
|  | α-pinene | 396 | 2.06 | 0.22 | ± | 0.07 | | 0.09 | ± | | 0.02 | |  |  |  |  |  |
|  | α-terpineol | 906 | 3.09 | 0.63 | ± | 0.13 | | 0.12 | ± | | 0.02 | | ****** |  |  |  |  |
|  | β-myrcene | 528 | 2.24 | 0.31 | ± | 0.3 | | 0.09 | ± | | 0.02 | | ******* |  |  |  |  |
|  | β-pinene | 486 | 2.43 | 0.16 | ± | 0.03 | | 0.08 | ± | | 0.01 | |  |  |  |  |  |
|  | limonene | 600 | 2.35 | 0.18 | ± | 0.01 | | 0.1 | ± | | 0.01 | | ****** |  |  |  |  |
|  | *trans*-α-bergamotene | 1302 | 2.39 | 0.2 | ± | 0.04 | | 0.07 | ± | | 0.01 | | ***** |  |  |  |  |
|  | *trans*-β-caryophyllene | 1272 | 2.57 | 0.02 | ± | 0.01 | | 0.03 | ± | | 0.01 | |  |  |  |  |  |
| **GLVs** | 1-hexanol | 276 | 2.6 | 0.28 | ± | 0.06 | | 0.29 | ± | | 0.07 | |  |  |  |  |  |
|  | *cis*-3-hexen-1-ol | 246 | 2.63 | 13.23 | ± | 2.47 | | 2.35 | ± | | 0.46 | | ****** |  |  |  |  |
|  | *cis*-3-hexenyl acetate | 570 | 2.95 | 1.17 | ± | 0.02 | | 0.16 | ± | | 0.03 | | ***** |  |  |  |  |
|  | *cis*-3-hexenyl butyrate | 912 | 2.7 | 0.54 | ± | 0.01 | | 0.06 | ± | | 0.02 | | ***** |  |  |  |  |
|  | *cis*-3-hexenyl formate | 384 | 2.99 | 0.01 | ± | 0.03 | | 0 | ± | | 0 | |  |  |  |  |  |
|  | *cis*-3-hexenyl isobutyrate | 834 | 2.61 | 0.98 | ± | 0.01 | | 0.05 | ± | | 0.01 | | ***** |  |  |  |  |
|  | *cis*-3-hexenyl propionate | 756 | 2.78 | 0.41 | ± | 0.25 | | 0.38 | ± | | 0.14 | |  |  |  |  |  |
|  | *cis*-3-hexenyl-2-methylbutanoate | 990 | 2.56 | 0.17 | ± | 0.00 | | 0.01 | ± | | 0.00 | | ***** |  |  |  |  |
|  | ethyl benzoate | 876 | 3.86 | 0.04 | ± | 0.08 | | 0.01 | ± | | 0.01 | |  |  |  |  |  |
|  | hexanal | 174 | 1.83 | 0.17 | ± | 0.06 | | 0.23 | ± | | 0.07 | |  |  |  |  |  |
|  | hexyl isobutyrate | 840 | 2.41 | 0.02 | ± | 0.03 | | n.d. | | | | |  |  |  |  |  |
|  | hexylbutyrate | 918 | 2.51 | 0.05 | ± | 0.03 | | 0.02 | ± | | 0.01 | | |  |  |  |  |
| **Aromatic compounds** | benzaldehyde | 462 | 4.74 | 0.2 | ± | 0.02 | | 0.16 | ± | | 0.03 | | |  |  |  |  |
|  | benzylalcohol | 624 | 4.48 | 0.05 | ± | 0.19 | | 0.05 | ± | | 0.01 | | |  |  |  |  |
|  |  |  |  |  |  |  | |  | | | | | |  |  |  | |
